# The Influence of High-Energy Milling on the Phase Formation, Structural, and Photoluminescent Properties of CaWO_4_ Nanoparticles

**DOI:** 10.3390/ma17153724

**Published:** 2024-07-27

**Authors:** Reni Iordanova, Maria Gancheva, Iovka Koseva, Peter Tzvetkov, Petar Ivanov

**Affiliations:** 1 Institute of General and Inorganic Chemistry, Bulgarian Academy of Sciences, Acad. G. Bonchev, Str. Bld. 11, 1113 Sofia, Bulgariaikossseva@svr.igic.bas.bg (I.K.); tzvetkov@svr.igic.bas.bg (P.T.); 2 Institute of Optical Materials and Technologies “Acad. Jordan Malinowski”, Bulgarian Academy of Sciences, 1113 Sofia, Bulgaria; petar@iomt.bas.bg

**Keywords:** high-energy ball milling, rapid synthesis, nanopowders, optical properties, emission spectra, CIE coordinates

## Abstract

CaWO_4_ nanoparticles were obtained by facile mechanochemical synthesis at room temperature, applying two different milling speeds. Additionally, a solid-state reaction was employed to assess the phase composition, structural, and optical characteristics of CaWO_4_. The samples were analyzed by X-ray diffraction (XRD), transition electron microscopy (TEM), and Raman, infrared (IR), ultraviolet–visible (UV–Vis) reflectance, and photoluminescence (PL) spectroscopies. The phase formation of CaWO_4_ was achieved after 1 and 5 h of applied milling speeds of 850 and 500 rpm, respectively. CaWO_4_ was also obtained after heat treatment at 900 °C for 12 h. TEM and X-ray analyses were used to calculate the average crystallite and grain size. The Raman and infrared spectroscopies revealed the main vibrations of the WO_4_ groups and indicated that more distorted structural units were formed when the compound was synthesized by the solid-state method. The calculated value of the optical band gap of CaWO_4_ significantly increased from 2.67 eV to 4.53 eV at lower and higher milling speeds, respectively. The determined optical band gap of CaWO_4_, prepared by a solid-state reaction, was 5.36 eV. Blue emission at 425 (422) nm was observed for all samples under an excitation wavelength of 230 nm. CaWO_4_ synthesized by the solid-state method had the highest emission intensity. It was established that the intensity of the PL peak depended on two factors: the morphology of the particles and the crystallite sizes. The calculated color coordinates of the CaWO_4_ samples were located in the blue region of the CIE diagram. This work demonstrates that materials with optical properties can be obtained simply and affordably using the mechanochemical method.

## 1. Introduction

Divalent metal tungstates with the general formula AWO_4_ (where A = Ca, Sr, Ba, Pb, Zn) have important scientific and industrial applications in various fields, such as scintillators in high-energy physics [1,2], laser and stimulated Raman-scattering active media [3], medical devices and drug delivery [4], catalysts [5,6,7], and luminescent materials [8,9,10,11]. Among the metal tungstates, calcium tungstate is a very attractive material due to its excellent thermal and chemical stability, as well as its good photovoltaic [12], electrical [13], magnetic [14], catalytic [5,15,16], and optical properties [7,8,9,17]. CaWO_4_ has a scheelite-type structure with the space group *I4_1_/a* and unit-cell parameters *a* = 5.243 Å and *c* = 11.373 Å [ICDD PDF#00-041-1431]. In this crystal structure, the alkaline ions are coordinated with eight oxygen atoms and form CaO_8_ polyhedra, and the tungstaten atoms are coordinated by four oxygen atoms in tetrahedral symmetry. The WO_4_ polyhedra are connected to CaO_8_ through common corners [18]. This compound is a semiconductor material with a wide band gap from 3.3 eV to 5.8 eV, which relates to different samples: bulk, nanoparticles, or film [5,9,12,13,19,20,21,22,23,24,25,26].

According to a number of studies, CaWO_4_ exhibits blue [4,10,11,24,25,26], blue-green [9,22], and green luminescence at room temperature [5,8,23,27]. Blue emission is commonly attributed to electronic transitions in the WO_4_ groups, while green originates from WO_3_ defect centers associated with oxygen vacancies. It is interesting to note that the particle shape of CaWO_4_ influences the kind and position of the emission of this compound. The CaWO_4_ with nano-rods and spherical morphology shows broad emission with maximum at 415–417 nm with different intensity [28]. This same crystal phase, with dumbbell, coral, rod, and dendrite particle morphologies, displays emission at 416 nm with various intensities and profiles [29]. The microspheres of CaWO_4_ exhibit emission at 420 nm, and the absolute PL intensity decreases with increasing temperature [30]. CaWO_4_ with a round morphology and an average size below 70 nm shows asymmetric blue-green emission at room temperature [31]. The aggregates of CaWO_4_ nano- and microcrystals possess intense luminescence emissions at 484 and 491 nm, respectively [22]. A broad emission peak at 521 nm was observed for CaWO_4_ with nearly spherical or elongated grains obtained by the molten salt method [7]. Emissions at 305 and 460 nm of CaWO_4_ with irregular particle morphology were described by Y. Wang et al., obtained by solvothermal synthesis [8]. 

Different physicochemical routes have been applied for the preparation of CaWO_4_, such as solid-state reactions [12,13,22], the Czochralski technique [2], hydrothermal synthesis [4,10,16,23], the sonochemical method [5,6], the molten salt method [8], solvothermal synthesis [9,28,31], co-precipitation [10,25], the double decomposition flux reaction method [32], electrospinning preparation [33], and mechanochemical synthesis [15,19,20,34]. 

Mechanochemical synthesis is one important and modern method for preparing nanomaterials in various fields from inorganic to metal–organic or organic materials [35,36,37,38]. Mechanical activation, as a combination of heat and pressure, causes different effects: defects and fresh surface areas that accelerate the synthesis of final products. The chemical reaction during the ball milling process depends on several factors, such as the nature of the initial reagents, the type of mills (horizontal rotary ball mill, planetary ball mill, vibration, shakier mill, etc.), milling speeds, milling time, the ball-to-powder weight ratio (BPR), and the milling environment (dry or wet). The milling speed has a decisive role for the chemical reaction at a constant BPR, the number and diameter of the balls, and the volume of the milling chamber [39,40,41,42]. Recently, mechanochemical treatment has been used for the preparation of optical materials due to the following advantages: an enhanced diffusion process, a short reaction time, and improved luminescent properties [43,44]. 

The literature data indicate that a lower milling speed has primarily been used for mechanochemical activation for the preparation of CaWO_4_. L. Cheng et al. used a lower speed (300/450 rpm) to prepare AWO_4_ (A = Ca, Ba, Sr) ceramics with good microwave dielectric properties [34]. P. Jena et al. obtained CaWO_4_ with a lower optical band gap after 12 h of mechanochemical treatment at 300 rpm and additional heat treatment at 1100 °C for 4 h [19]. S. Balamurugan et al. reported a thorough investigation of the phase formation of CaWO_4_ at a 300 rpm milling speed using various amounts of reaction mixtures with varying numbers and diameters of WC balls, under dry and wet conditions [20]. The authors obtained the pure phase of CaWO_4_ after 10 h of milling time in a dry atmosphere. Keeping in mind the above-mentioned literature data, as well as our previous results for the direct mechanochemical synthesis of similar inorganic compounds, we chose 2.5 g of reaction mixture (CaCO_3_ + WO_3_) and applied two grinding speeds, 500 and 850 rpm, to produce CaWO_4_. 

The aim of the present work is to verify the possibility of direct synthesis in a short reaction time at a higher grinding rate. To the best of our knowledge, the influence of high-energy ball milling on the photoluminescent properties of CaWO_4_ has not been reported. The structural and luminescent properties obtained are compared with those of CaWO_4_ obtained by solid-state reaction.

## 2. Materials and Methods

### 2.1. Direct Mechanochemical Synthesis

The reagents used in the mechanochemical treatment were CaCO_3_ (Merck KGaA, Amsterdam, The Netherlands, 99.9% purity) and WO_3_ (Merck KGaA, Amsterdam, The Netherlands, 99.9% purity). The stoichiometric ratio of the initial materials was 1:1, which corresponds to the crystal CaWO_4_ phase. The high-energy ball milling of the initial mixture was carried out in a planetary ball mill (Fritsch premium line, Pulverisette No 7, FRITSCH GmbH, Idar-Oberstein, Germany), where two different milling speeds were applied: 500 and 850 rpm. The activation was performed in air atmosphere, and the ball-to-powder weight ratio was 10:1. To minimize the temperature during milling, the process was carried out in periods of 15 min, with rest periods of 5 min according to our previous studies [15,39,40,41]. The labels of the milled samples were as follows: CaWO_4_-I for the milling speed of 500 rpm; CaWO_4_-II for the milling speed of 850 rpm.

### 2.2. Solid-State Reaction 

The initial reagents for the solid-state reaction were identical to those used in the mechanochemical activation i.e., CaCO_3_ (Merck KGaA, Amsterdam, The Netherlands, 99.99%) and WO_3_ (Merck KGaA, Amsterdam, The Netherlands, 99.99%). The stoichiometric ratio was the same (1:1). The initial mixture was homogenized in an agate mortar. Subsequently, the mixture was transferred to an aluminum crucible and heated at 900 °C for 12 h in an electrical furnace. The heat treatment was carried out according to the data in reference [13]. The prepared sample was labeled CaWO_4_-III. 

### 2.3. Characterization 

The XRD powder patterns were collected using the Bruker D8 Advance X-ray powder diffractometer, Karlsruhe, Germany, equipped with a CuKa radiation source (1.542 A) and the LynxEye PSD detector. The measurement range was from 5.5° to 120.0° 2θ, with a step of 0.02° 2θ and a counting time of 1.0 s/strip (for a total of 175.0 s/step, according to the PSD detector). The qualitative phase analysis was performed using the DIFFRAC.EVA v.4 software program [45] and the ICDD PDF-2 (2021) reference database. The unit cell parameters and crystallites size were calculated using the whole profile of the powder pattern and Topas v.4.2 software [46]. TEM observation was performed by a JEOL JEM-2100 microscope (Akishima, Japan) at an accelerating voltage of 200 kV. The preparation procedure of the specimens consisted of dispersing them in ethanol by ultrasonic treatment and then dripping them onto standard Cu grids. The Raman spectra were recorded using the Via Qontor Raman Confocal Microscope (Renishaw plc, Wotton-under-Edge, UK) with a laser wavelength of 532 nm (Nd:YAG-Laser). The laser power on the sample was maintained at 1% of the nominal power, so no heating effects on the powder sample could be observed. The excitation light was focused and collected using a ×50 LWD objective lens. Infrared spectra were registered in the range of 1200–400 cm^−1^ on a Nicolet-320 FTIR spectrometer (Madison, WI, USA) using the KBr pellet technique with a spectral resolution of 2 nm. The diffuse-reflectance spectra were recorded with a Thermo Evolution 300 UV-Vis Spec-trophotometer equipped with a Praying Mantis device (Madison, WI, USA) with Spectralon reflectance standard. For recording the background, Spectralon was used. The PL emission spectra were measured on a Horiba Fluorolog 3-22 TCS spectrophotometer (Longjumeau, France) equipped with a 450 W Xenon Lamp as the excitation source. The automated modular system, with the highest sensitivity among those available on the market, was used, allowing for the measurement of light emission of practically any type of sample. Double-grating monochromators were used for emissions in the range of 200–950 nm. All spectra were measured at room temperature.

## 3. Results and Discussion 

### 3.1. Phase Formation and Morphology

#### 3.1.1. X-ray Powder Diffraction Analysis

**Figure 1:**
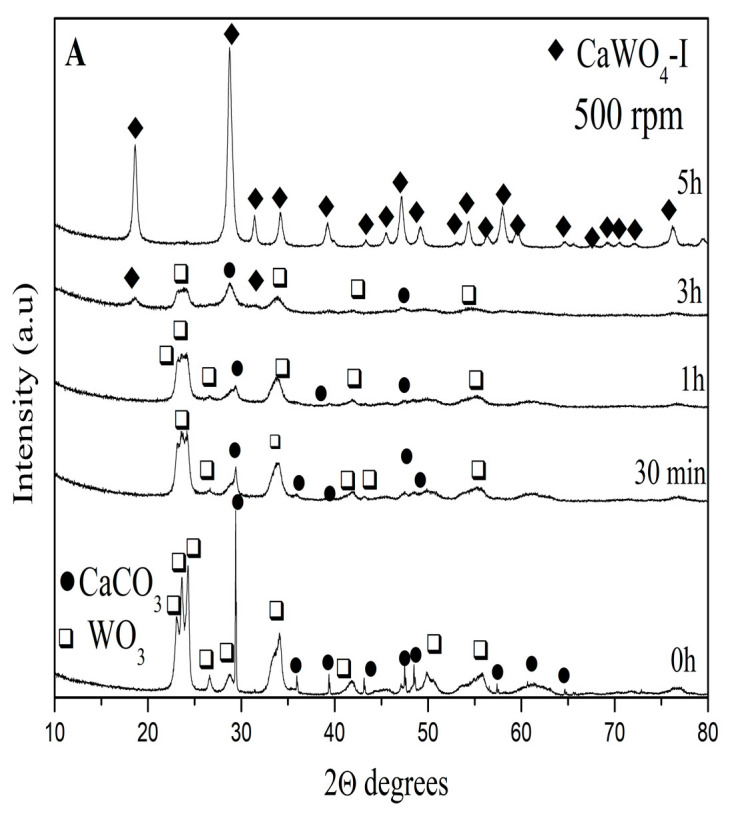

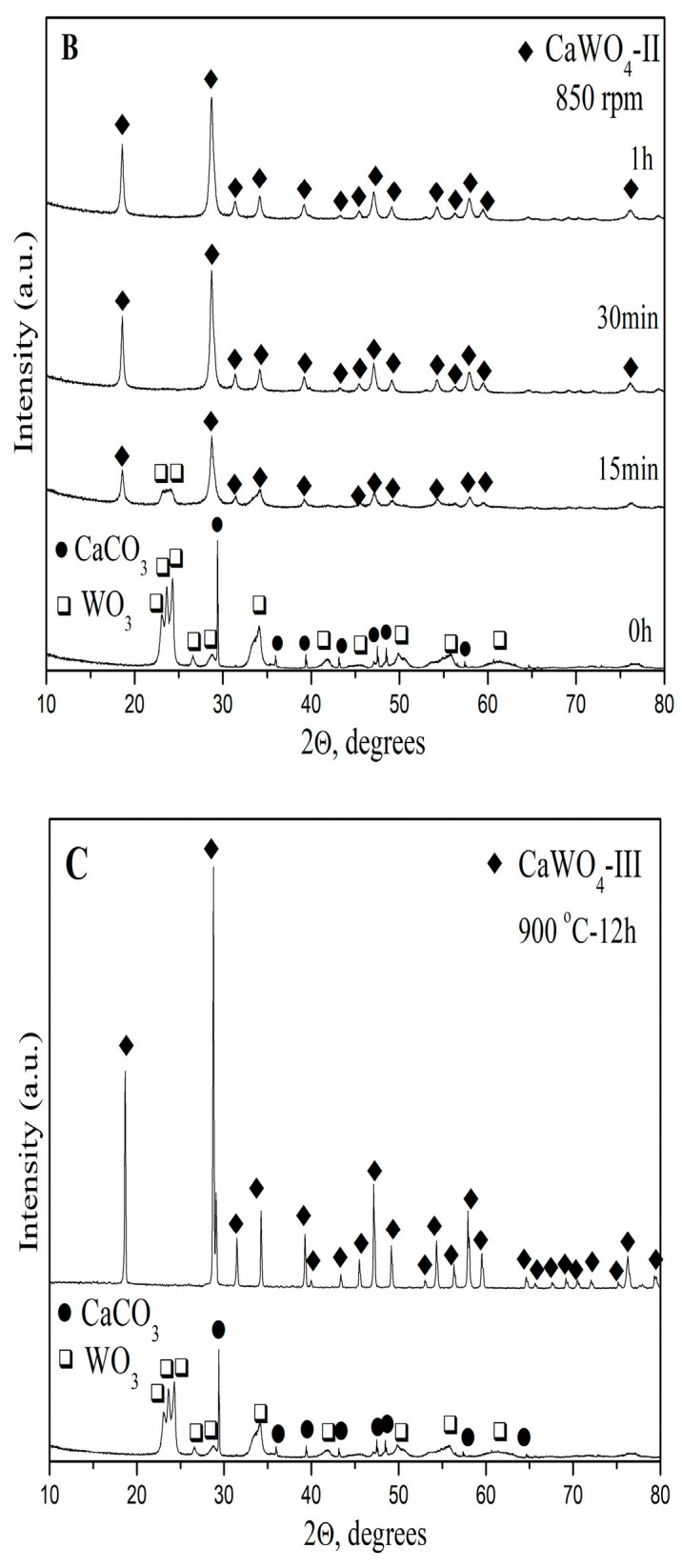
XRD patterns of the initial mixture and mechanochemically activated sample at 500 rpm (**A**), mechanochemically activated sample at 850 rpm (**B**), and CaWO_4_ obtained after solid-state reaction (**C**).

**Figure 2:**
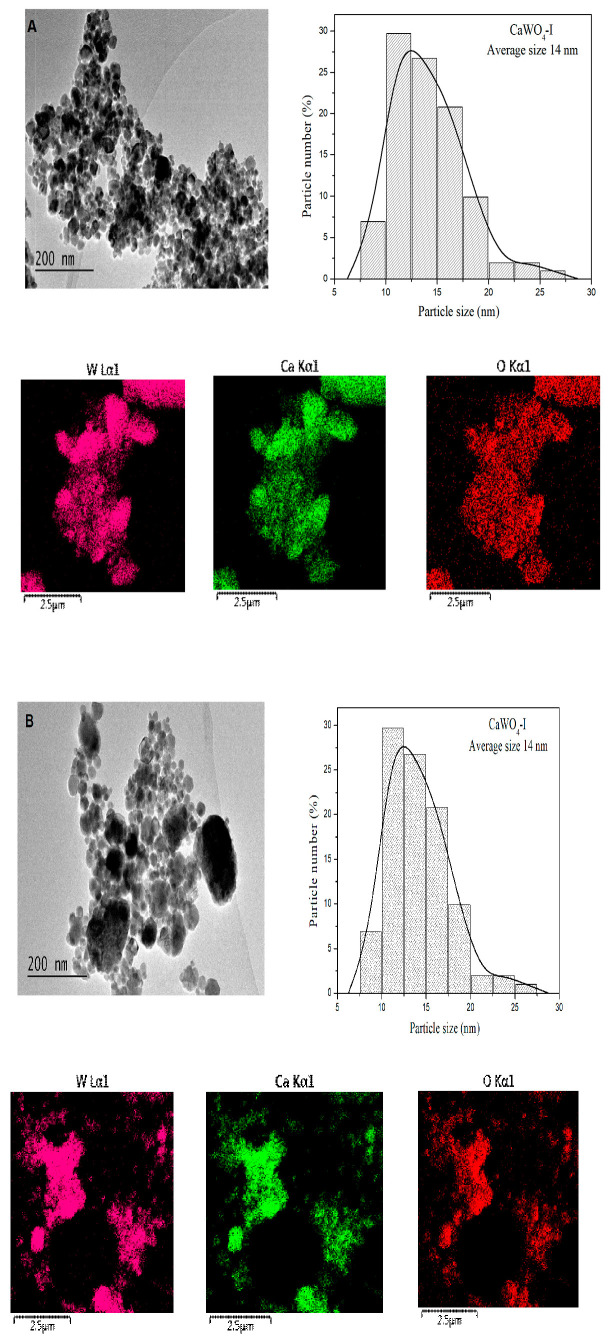

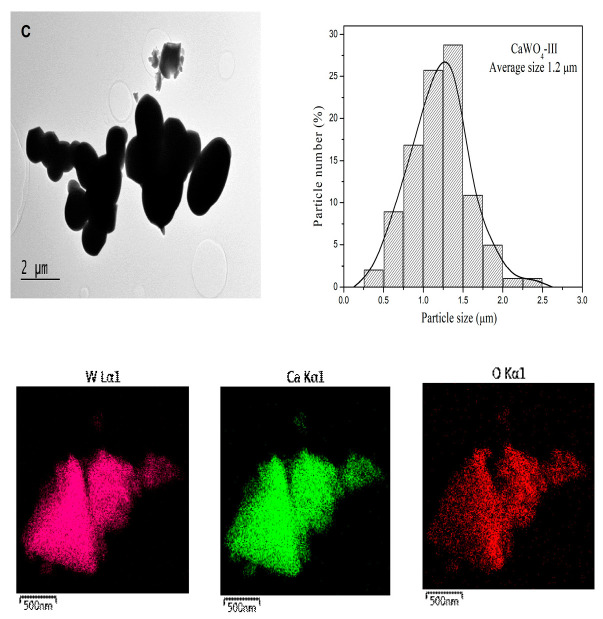
(**A**) TEM image, particle size distribution, and EDS mapping of CaWO_4_-I obtained after 5 h of milling time at 500 rpm. (**B**) TEM image, particle size distribution, and EDS mapping of CaWO_4_-II obtained after 1 h of milling time at 850 rpm. (**C**) TEM image, particle size distribution, and EDS mapping of CaWO_4_-III obtained by solid-state reaction.

A comparative analysis of the phase formation, morphology, and symmetry of the structural units and optical properties of the mechanochemically and solid-state-obtained CaWO_4_ powders was carried out. The influence of the milling speed on the reaction time, crystallite size, and defects in the CaWO_4_ was studied by X-ray diffraction analysis (Figure 1A,B). The XRD pattern of the initial mixture shows the principal peaks of the monoclinic WO_3_ (PDF# 01-072-0677) and the orthorhombic CaCO_3_ (PDF# 00-041-1475). The milling speed of 500 rpm led to a decrease in the intensity and the broadening of all diffraction lines after 1 h of milling time (Figure 1A). This was a result of a decrease in the particle size, destruction of the long-range order, and the partial amorphization of both reagents. Increasing the milling time to 3 h caused the appearance of reflections characteristic for tetragonal CaWO_4_ (PDF#00-041-1431). This is an indication of the beginning of the chemical reaction between the activated reagents. The reaction finished after 5 h of milling time (Figure 1A). The possibility of phase formation after a short time was checked using a higher milling speed of 850 rpm. In this case, the principal peaks of CaWO_4_ were observed after 15 min of milling time. But the diffraction lines typical of the initial WO_3_ at 2θ = 23.20 and 23.95° were detected (Figure 1B). The pure CaWO_4_ was synthesized after 30 min of milling time, which is shorter compared to the synthesis at the 500 rpm milling speed. The increase in the milling time to 60 min did not affect the phase composition. In both cases, the diffraction lines were broad, which was attributed to the smaller size of the mechanochemically synthesized CaWO_4_ particles. The TEM images of these samples also show the formation of particles with lower dimensions (Figure 2A,B). Compared to the research reported in Ref. [20], we found that the amount of 2.5 g of the reaction mixture, higher milling speeds of 500 and 850 rpm, and dry media were more suitable conditions for the rapid preparation of CaWO_4_. Figure 1C exhibits the XRD pattern of CaWO_4_ after heat treatment at 900 °C for 12 h. A remarkable narrowing of the diffraction lines was observed due to the higher crystallinity of the CaWO_4_ in comparison to the mechanochemically obtained CaWO_4_ powders. No additional diffraction lines were found, meaning that the obtained sample was pure single phase. The lattice parameters (a, c, unit cell), lattice strain, and crystallite size of the CaWO_4_ obtained from the XRD refinement are shown in Table 1.

Table 1 presents the calculated unit cell parameters, crystallite sizes, and isotropic microstrains of the three synthesized samples. These were compared with data published in the literature [18]. It is clear from the values that there was no significant change in the parameters or volumes of the unit cells of the CaWO_4_ regardless of the conditions and method of preparation. Quite expectedly, as the mechanochemical activation energy increased, the crystallite size of the treated sample decreased. This was particularly clearly observed in the obtained values for the crystal lattice microstrains, which almost doubled when the milling speeds increased from 500 rpm to 850 rpm. The lattice strain decreased with an increasing crystallite size due to the prolongation of the heat treatment at a high temperature (CaWO_4_-III). 

#### 3.1.2. TEM Analysis

The size and morphology of the particles are important factors for the optical application of CaWO_4_. Figure 2A–C presents the TEM images, particle size distribution, and EDS mapping of the investigated samples. The particles of CaWO_4_-I and II obtained by mechanochemical treatment (milling speeds of 500 and 850 rpm) are well separated from each other, with a quasihexagonal form (Figure 2A,B). The size histograms illustrate that both CaWO_4_ nanoparticles exhibited good dispersion and a narrower size distribution. The particle size distribution of the CaWO_4_-I obtained at the lower milling speed of 500 rpm was between 5 and 30 nm, with most particles in the range between 10 and 15 nm (Figure 2A). At the higher milling speed, the particles had an average size of 20 nm (Figure 2B). Agglomeration of the particles was not observed in either of the mechanochemically synthesized samples. As might be expected, the particles obtained by solid-state synthesis were much larger, with an oval shape and average particle size of 1200 nm (Figure 2C). The particle distribution was between 500 and 2500 nm, with most particles tending to be between 1000 and 1500 nm in size. In this case, the agglomeration of the particles was visible. We can infer from the TEM results that significantly smaller particles were produced by high-energy ball milling. The EDS mapping demonstrates that the elements Ca, W, and O were disseminated on the entire surface of the samples prepared by mechanochemical activation and solid-state reaction.

### 3.2. Raman and Infrared Spectroscopy

**Figure 3:**
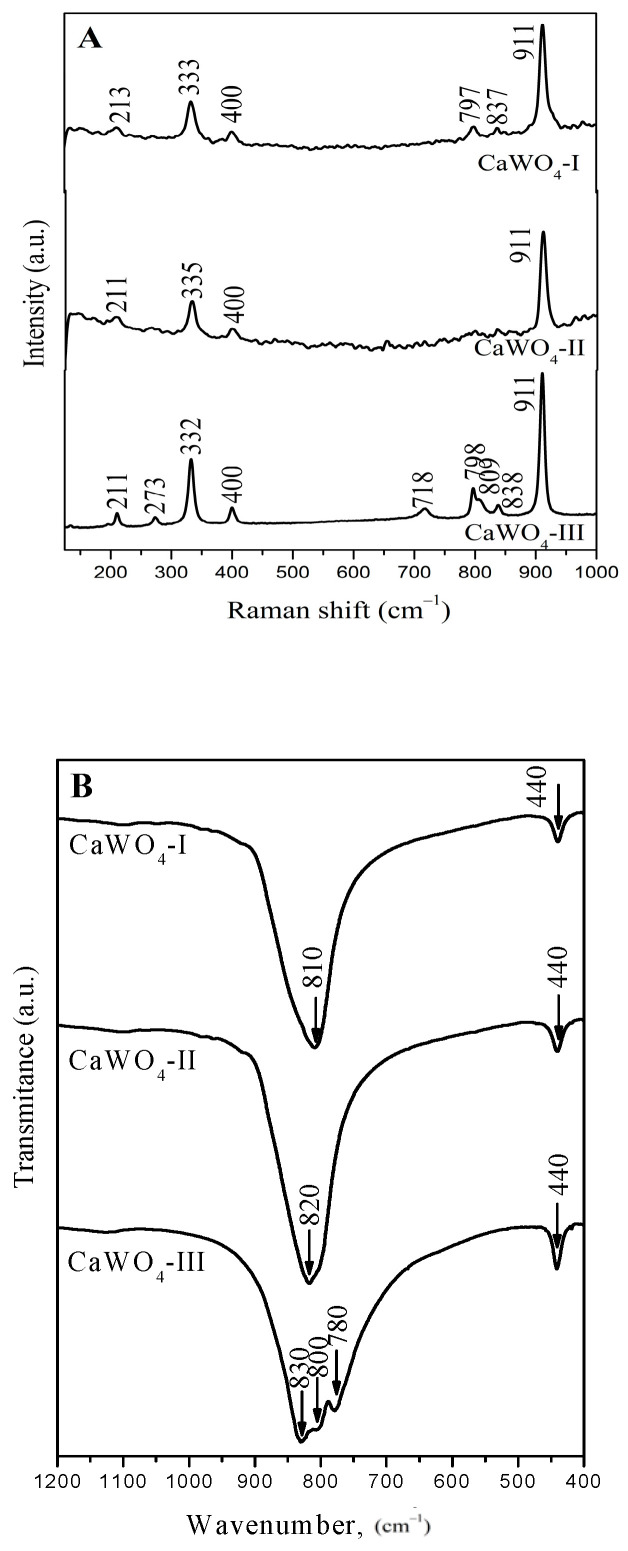
(**A**) Raman spectra of the samples synthesized by mechanochemical treatment (CaWO_4_-I, 5 h at 500 rpm; CaWO_4_-II, 1 h at 850 rpm) and solid-state reaction (CaWO_4_-III). (**B**) Infrared spectra of the samples synthesized by mechanochemical treatment (CaWO_4_-I, 5 h at 500 rpm; CaWO_4_-II, 1 h at 850 rpm) and solid-state reaction (CaWO_4_-III).

Both types of spectroscopic spectra of the obtained products evidence the formation of CaWO_4_ with a scheelite type structure with different degrees of WO_4_ destruction. Figure 3A shows the Raman spectra of the CaWO_4_ phase excited at 532 nm (Nd:YAG-Laser). The Raman bands can be divided into two groups—internal and external modes—due to the weak coupling between the WO_4_ and CaO_8_ groups and keeping in mind the literature data [5,18,23,47]. The internal modes are attributed to the oscillations inside the [WO_4_]^2−^ units with an immovable mass center. The external or lattice phonons correspond to the motion of the Ca^2+^cations and WO_4_ units [47,48]. 

The spectrum of CaWO_4_-III produced by the solid-state reaction was used to assign the Raman peaks: the strong band at 911 cm^−1^ was due to the symmetric stretching *ν*_1_ of W–O bond in the WO_4_ tetrahedra [5,18,23,47,48]. The appearance of several absorption bands in the range of 838–718 cm^−1^ can be attributed to the elimination of the degeneracy of the *ν*_3_ vibration of different crystallographically nonequivalent WO_4_ tetrahedra with different local symmetry (T_d_, C_3_, C_2v_) [49]. The observed band at 332 cm^−1^ and the weak band at 400 cm^−1^ are the result of the *ν*_2_ vibrations of the W-O bond [47,48]. The low frequency bands at 273 and 210 cm^−1^ could be assigned to the translational mode of the ν(Ca–O) and the bending vibration of the [WO_4_]^2−^ group in the CaWO_4_, respectively [23,50]. The absence of the peaks in the range from 840 to 400 cm^−1^ in the CaWO_4_-II sample after 1 h of milling time at 850 rpm indicates the generated WO_4_ units were more symmetric compared to the other samples (CaWO_4_-I and CaWO_4_-III). These results were confirmed by the infrared (IR) spectroscopy (Figure 3B). The IR spectra of the CaWO_4_-I and CaWO_4_-II obtained by mechanochemical synthesis display one absorption band around 810 (820) cm^−1^ due to the *ν*_3_ vibration of the WO_4_ structural unit building the crystalline structure of the CaWO4 [15,17,25]. The low-intensity band at 440 cm^−1^ is attributed to the *ν*_4_ modes of the same structural groups [51]. In contrast, the IR spectrum of the CaWO_4_-III prepared by solid-state reaction exhibited more absorption bands between 900 and 700 cm^−1^. In this case, the appearance of the bands at 830, 800, and 780 cm^−1^ are assigned to the elimination of the *ν*_3_ vibration degeneracy of the WO_4_ tetrahedral with a lower local symmetry [52]. Similar results were established for BaMoO_4_ obtained by solid-state reaction [41]. The results above show that more symmetrical WO4 structural units were formed due to the high-energy ball milling (Figure 3B).

### 3.3. Optical Properties

#### 3.3.1. UV–Vis Absorbance Spectroscopy

**Figure 4:**
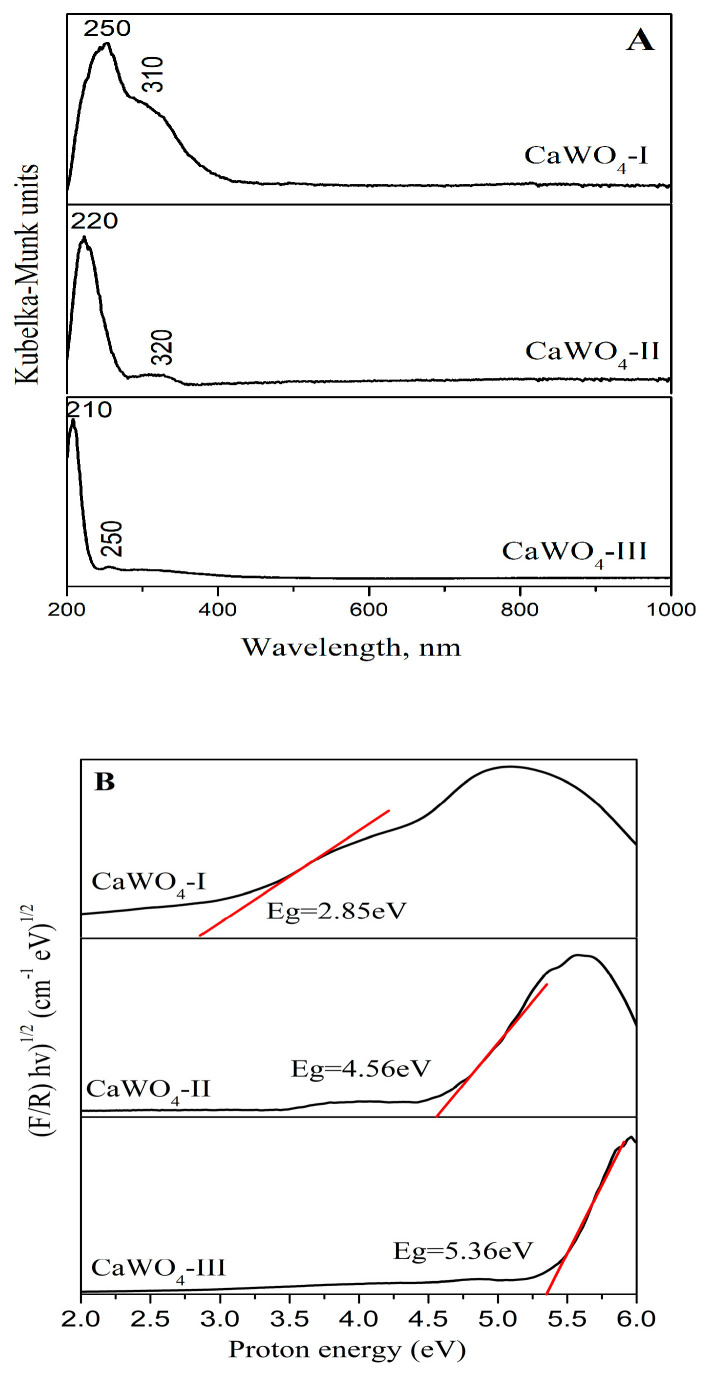
(**A**) UV-Vis spectra of the samples obtained by mechanochemical synthesis treatment (CaWO_4_-I, 5 h at 500 rpm; CaWO_4_-II, 1 h at 850 rpm) and solid-state reaction (CaWO_4_-III). (**B**) Tauc’s plot of the samples obtained by mechanochemical synthesis treatment (CaWO_4_-I, 5 h at 500 rpm; CaWO_4_-II, 1 h at 850 rpm) and solid-state reaction (CaWO_4_-III).

**Figure 5:**
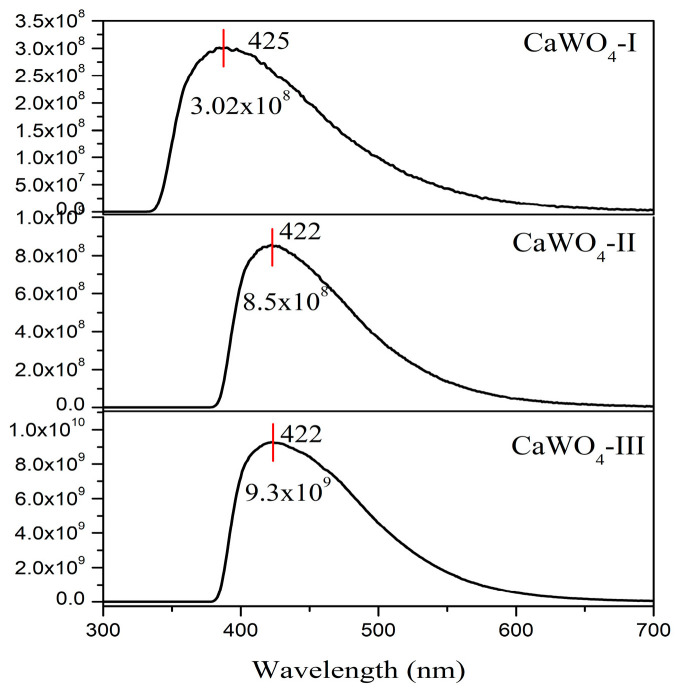
Emission spectra of samples obtained by mechanochemical treatment (CaWO_4_-I, 5 h at 500 rpm; CaWO_4_-II, 1 h at 850 rpm) and solid state reaction (CaWO_4_-III).

**Figure 6:**
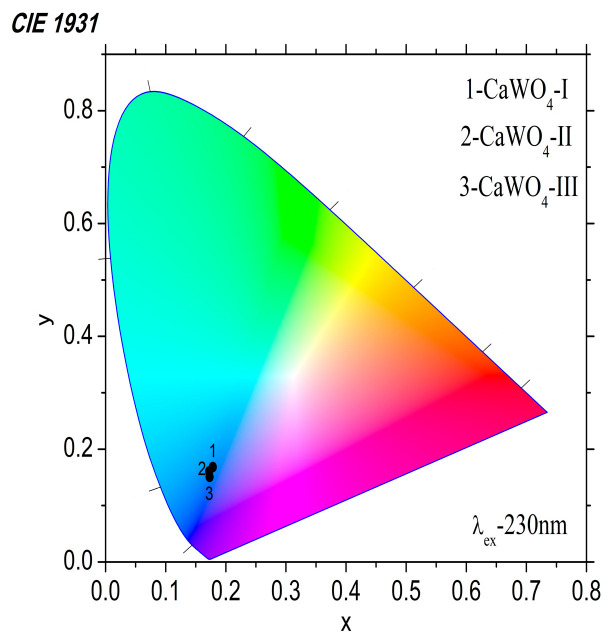
CIE chromaticity diagram of samples obtained by mechanochemical treatment (CaWO_4_-I, 5 h at 500 rpm; CaWO_4_-II, 1 h at 850 rpm) and solid-state reaction (CaWO_4_-III).

The optical behavior of the CaWO_4_ powders obtained by both techniques was investigated using UV-Vis (Figure 4A,B) and photoluminescence spectroscopies (Figure 5 and Figure 6). The UV–visible absorption spectra was transformed in the Kubelka–Minck function using the following formula [F(R)]: F(R∞) = (1 − R∞)^2^/(2R∞) = K/S. The R is the diffuse reflectance of the sample, K is the absorption coefficient, and S is the scattering coefficient. The reflectance of the sample depends on the ratio of K to S but not on the absolute values of K and S [53,54]. A strong absorption in the range of 210–250 nm was observed for all obtained samples (Figure 4A). The position of the main peak depends on the processing conditions i.e., the milling speeds (CaWO_4_-I and CaWO_4_-II) and the solid-state method (CaWO_4_-III). In the UV-Vis spectrum, the CaWO_4_-I sample exhibited a wide range from 210 to 370 nm with a maximum peak at 250 nm. The absorption band shifted up to 220 nm when the milling speed was increased to 850 rpm. CaWO_4_-III, obtained by solid-state reaction, also showed a tendency toward a blue shift, with a notable absorption peak recorded at 210 nm (Figure 4A). The narrowest absorption line was detected for this sample. This fact can be attributed to the higher crystallinity nature of the solid-state reaction-obtained powder with the highest crystalline size (D = 370 nm). The bands between 210 and 250 nm were attributed to the charge-transfer transitions within the WO_4_^2−^ complex in the scheelite type inorganic phases [5,7,17,24,25,30]. In addition, a secondary absorption band between 250 and 320 nm was also visible. Keeping in mind the reported data, this band corresponds to the creation of the excitonic state in A^2+^ ions (A^2+^ = Ba, Sr, Ca) [33,55]. A similar UV-Vis spectra was reported for CaWO_4_ obtained by different methods, including the sonochemical method [5], solvothermal method [9,23], solid-state reaction [12,13], and hydrothermal route [16].

The calculated Eg values of the CaWO_4_ powders obtained by mechanochemical activation at 500 and 850 rpm were 2.88 and 4.59 eV, respectively. The optical band gap value of CaWO_4_-I (5 h, 500 rpm) is similar to those of CaWO_4_ obtained by 12 h of milling time and heat-treated at 1100 °C [19]. The calculated optical band gap of CaWO_4_-III produced by the solid-state technique is 5.27 eV (Figure 4B). This increase in the band gap is a result of the structural improvements, according to the XRD analysis. The value is close to that of the CaWO_4_ thin film obtained by the chemical solution method [21] and the CaWO_4_ powders obtained by solid-state reaction [13], the polymeric precursor method, and the microwave-assisted hydrothermal method [56]. The changes in the crystallite size and microstructure of the CaWO_4_ obtained by both preparation methods influenced the electron structure, and this is reason for the difference in value of the optical band gaps and their optical features [57]. 

#### 3.3.2. Luminescent Properties

Figure 5 compares the emission spectra of the CaWO_4_ synthesized by both procedures: mechanochemical activation (500 and 850 rpm) and solid-state reaction (900 °C for 12 h). All samples show a broad blue band with a maximum at around 422–425 nm upon excitation at 230 nm (absorption of the WO_4_ groups). The emission lines show no variation in shape, but there is a noticeable difference in the emission intensity. The emission peaks of all prepared CaWO_4_ are in good agreement with the literature data for blue emission [58,59]. In our case, although the obtained crystalline phases possessed different particle size and morphologies, the emission spectrum profiles were the same. The blue emission at 425 nm, with a full width at the half maximum of 98 nm, was registered for CaWO_4_-I obtained using the lower milling speed (500 rpm), while the blue emission was changed at 422 nm, and a lower full width at the half maximum for CaWO_4_-II was obtained after the short milling time (1 h) at 850 rpm. The same blue maximum emission (422 nm) was observed with a full width at the half maximum of 100 nm for the solid-state-synthesized sample (CaWO_4_-III). This demonstrates that the as-prepared broader blue CaWO_4_ materials are promising candidates for WLEDs.

The PL intensities at 425–422 nm gradually increased with an increasing crystallite size. The value of the emission intensity was: ~3.02 × 10^8^ for the CaWO_4_-I prepared after 5 h of milling time and a milling speed of 500 rpm; ~8.5 × 10^8^ was recorded for the CaWO_4_-II obtained after a 1 h milling time and a milling speed of 850 rpm; ~9.3 × 10^9^ was recorded for the CaWO_4_-III obtained by the solid-state technique. The highest blue emission of CaWO_4_-III was probably due to its good crystallinity, oval-shaped particles, and highly asymmetric WO_4_ structural units. Similar results regarding the emission intensity were discussed by F. Lei et al. [60]. According to the literature data, the PL intensity and emission behavior of MWO_4_ with the scheelite and wolframite type structure depends on the surface chemistry, morphology, and particle size [61]. The lower crystallite size that results from mechanochemical treatment may be the cause of the lowest blue emissions for CaWO_4_-I and CaWO_4_-II [62]. The Commission International de I’Eclairage (CIE)’s chromaticity diagram of the CaWO_4_ samples excited at 230 nm is shown in Figure 6. As the main peak in the luminescence spectra is in the range between 400 and 600 nm and peaks at 425 (422) nm, it is expected that the emitted light will be blue. The calculated chromaticity coordinates are very close values and fall into the blue range on the CIE diagram (Figure 6 and Table 2). Blue light is in high demand because it can be mixed with yellow or participate in the tricolor (red, green, blue) to produce white light.

## 4. Conclusions

The variation in the milling speed promoted the rapid synthesis of the CaWO_4_ from 5 h at 500 rpm to 1 h at 850 rpm without additional heat treatment. We demonstrated that the mechanochemical treatment led to the formation of particles with a quasihexagonal form and a narrower size distribution. CaWO_4_ was also obtained by solid-state reaction at 900 °C for 12 h. Agglomeration of the oval particles was obtained using the solid-state technique. It was established that the method of preparation influences the crystallinity nature, macrostrains, and the symmetry of the WO_4_ entity and its optical properties. It was established that the optical band gap of CaWO_4_ strongly depends on the milling conditions, i.e., an Eg increase from 2.85 eV to 4.59 eV with an increase in the milling speed. The CaWO_4_-III obtained by the solid-state reaction possessed higher crystallinity, asymmetric WO_4_ units, and oval particles, which resulted in a higher blue emission intensity compared to the mechanochemically synthesized samples.

## Figures and Tables

**Table 1:** Unit cell parameters, crystallite size, and lattice microstrain values of the obtained samples.

	CaWO_4_-I 5 h, 500 rpm	CaWO_4_-II 1 h, 850 rpm	CaWO_4_-III 900 °C-12 h	CaWO_4_ (PDF#00-041-1431)
Unit cell parameters a/c (Å)	5.244(1) 11.388(2)	5.242(1) 11.375(2)	5.238(1) 11.377(2)	5.243 11.373
Unit cell volume (Å^3^)	313.19(1)	312.598(3)	312.22(1)	312.63
Crystallites size (nm)	31.5(1)	24.4(7)	369(5)	n.a.
Lattice strain ε_0_ × 10^−4^	6.92(10)	12.55(8)	2.067(15)	n.a.

**Table 2:** Emission peaks, FWHM, and CIE coordinates (x, y) of the synthesized samples.

Samples	Emission Peak, nm	FWHM, nm	x	y
CaWO_4_-I, 5 h at 500 rpm	425	98	0.178	0.168
CaWO_4_-II 1 h at 850 rpm	422	92	0.173	0.150
CaWO_4_-III 900 °C for 12 h	422	100	0.172	0.161

## Data Availability

The data are contained within the article.
